# The *Feline calicivirus* Leader of the Capsid (LC) Protein Contains a Putative Transmembrane Domain, Binds to the Cytoplasmic Membrane, and Exogenously Permeates Cells

**DOI:** 10.3390/v16081319

**Published:** 2024-08-19

**Authors:** Yoatzin Peñaflor-Téllez, Jesús Alejandro Escobar-Almazan, Carolina Pérez-Ibáñez, Carlos Emilio Miguel-Rodríguez, Jaury Gómez de la Madrid, Erick I. Monge-Celestino, Patricia Talamás-Rohana, Ana Lorena Gutiérrez-Escolano

**Affiliations:** Departamento de Infectómica y Patogénesis Molecular, Centro de Investigación y de Estudios Avanzados del IPN, Av. IPN 2508, Col. San Pedro Zacatenco, Mexico City 07360, Mexico; yp482@njms.rutgers.edu (Y.P.-T.); jesus.escobar@cinvestav.mx (J.A.E.-A.); carolina.perez@cinvestav.mx (C.P.-I.); carlos.miguel@cinvestav.mx (C.E.M.-R.); jaury.gomez@cinvestav.mx (J.G.d.l.M.); erick.monge@cinvestav.mx (E.I.M.-C.); ptr@cinvestav.mx (P.T.-R.)

**Keywords:** FCV, viroporins, LC, membrane permeation

## Abstract

*Feline calicivirus* (FCV), an important model for studying the biology of the *Caliciviridae* family, encodes the leader of the capsid (LC) protein, a viral factor known to induce apoptosis when expressed in a virus-free system. Our research has shown that the FCV LC protein forms disulfide bond-dependent homo-oligomers and exhibits intrinsic toxicity; however, it lacked a polybasic region and a transmembrane domain (TMD); thus, it was initially classified as a non-classical viroporin. The unique nature of the FCV LC protein, with no similarity to other proteins beyond the *Vesivirus* genus, has posed challenges for bioinformatic analysis reliant on sequence similarity. In this study, we continued characterizing the LC protein using the AlphaFold 2 and the recently released AlphaFold 3 artificial intelligence tools to predict the LC protein tertiary structure. We compared it to other molecular modeling algorithms, such as I-Tasser’s QUARK, offering new insights into its putative TMD. Through exogenous interaction, we found that the recombinant LC protein associates with the CrFK plasmatic membrane and can permeate cell membranes in a disulfide bond-independent manner, suggesting that this interaction might occur through a TMD. Additionally, we examined its potential to activate the intrinsic apoptosis pathway in murine and human ovarian cancer cell lines, overexpressing survivin, an anti-apoptotic protein. All these results enhance our understanding of the LC protein’s mechanism of action and suggest its role as a class-I viroporin.

## 1. Introduction

Since its isolation in 1957, *feline calicivirus* (FCV) has been used as a model for studying the biology of the members of the *Caliciviridae* family [1]. Due to its ability to replicate efficiently in cell culture in a cost-effective manner, alongside the existence of various reverse-genetic systems, this virus has proven invaluable in elucidating the strategies employed by members of this viral family to replicate and cause diseases of medical and veterinary importance. While the family members share proteins with similar activities, such as VPg, the protease (NS6), and the replicase (NS7), some members encode unique proteins, such as virulence factor 1 (VF1), an important immune response and apoptosis modulator found thus far only in *Murine norovirus* (MNV). Similarly, the leader of the capsid (LC) is a unique protein of the *Vesivirus* genus members, which do not exhibit homology with other proteins reported in databases. Encoded in the subgenomic RNA, the LC-VP1 precursor protein is processed by the protease–polymerase NS6/7 into the LC and VP1 mature forms [2]. The 14 kDa LC protein is composed of 124 amino acids and contains two conserved regions (CRs): a poly-cysteine (CR-I) in the N-terminus and a poly-proline (CR-II) in the C-terminus. The FCV LC protein is responsible for the cytopathic effect (CPE) induction. It is essential for virus replication, as demonstrated by alanine substitutions of the cysteines in the CR-I. For example, substitutions, such as those in the C40A mutant (C40A mut) LC protein, resulted in the loss of the cell rounding phenotype characteristic of the CPE and the ability of the virus to spread efficiently [3]. Serial passages of the FCV containing this mutation resulted in compensatory mutations that restored the CPE, which indicates a strong selective pressure to reestablish the LC protein function in CPE [3]. Our workgroup has found that the LC protein expression in a virus-free system causes the degradation of two inhibitors of apoptosis proteins (IAPs), survivin and XIAP, and induces apoptosis [4]. Through in silico and in vitro assays, we determined that this viral protein exhibits viroporin characteristics, such as its ability to homo-oligomerize and its toxicity through osmotic stress induction in a heterologous system [5]. We have recently found that the FCV LC protein is located on the inner and outer faces of the plasma membrane of infected cells, and in the extracellular media, suggesting that it is secreted [6].

Although its poor protein sequence homology poses a challenge for traditional modeling tools, the recent use of artificial intelligence servers such as AlphaFold and its successors AlphaFold 2 and 3, which enables reliable 3D structure prediction ab initio—without templates of other proteins whose structure has been determined experimentally—can help to resolve this issue [7]. AlphaFold 2, for example, has been successfully used to predict the tertiary structure of viral proteins that exhibit poor homology in their sequences, such as those from SARS-CoV-2 and monkeypox, representing an important tool for predicting their function [7,8].

In this work, we conducted a bioinformatic analysis using the AlphaFold 2 predictor to determine the structure of the FCV LC protein as a starting point to compare our findings with bioinformatic analyses previously reported [5] and to predict a potential TMD confidently. We found the presence of a C-terminal TMD, which was redundantly confirmed in its sequence by the updated predictor of TMDs PSI-PRED. Additionally, the predicted structure of the LC protein shows a putative insertion into a modeled mammalian plasma membrane through this putative TMD. The ability of the recombinant LC protein to reach the plasmatic membrane of CrFK cells was demonstrated by its exogenous interaction in non-permeabilized cells, which is in concordance with the in silico results. Moreover, the wild-type LC protein could permeate the membrane of CrFK cells in an exogenous interaction, a common characteristic of viroporins. Both the ability of the FCV LC protein to permeate the cellular plasma membrane and to induce apoptosis also occurred in murine and human ovarian cancer cell lines overexpressing survivin, highlighting its potential role in oncological research and as a therapeutic tool.

## 2. Materials and Methods

### 2.1. Prediction and Validation of the Tertiary Structure of the WT and C40A Mutant LC Proteins

The sequence of the LC protein from the FCV Urbana strain was obtained from NCBI protein (accession Q66915.1) and submitted to AlphaFold 2 (DeepMinds 41586-021-03819-2) and AlphaFold 3 [9] or tertiary structure prediction through the Google Collab online notebook and standard prediction conditions. The highest score models for both WT and C40A mut LC proteins were analyzed with MolProbity [10] to determine the Z-score and obtain Ramachandran plots. Model snapshots were obtained with PyMol (DeLano Scientific LLC, San Francisco, USA), highlighting the putative TMD in red.

### 2.2. Prediction of the Transmembrane Domain of the WT and C40A Mutant LC Proteins

The sequence of the LC protein from the FCV Urbana strain was used as input with the Es PSIPRED 4.0 MEMSAT-SVM software predictor under standard conditions. Tertiary structure models of the WT and C40A mut LC proteins, obtained and validated as previously described, were used for modeling the putative model in the membrane with the PPM 3.0 web server [11] using a mammalian, non-curved plasma membrane, with the N-terminus oriented to the inner face of the membrane. The resulting models were visualized with PyMol. The putative transmembrane helices are highlighted in red.

### 2.3. Cell Culture

Crandell Reese Feline Kidney cells (CrFK/CCL-94) obtained from the American Type Culture Collection (ATCC) and ID8 cells obtained from Sigma (Sigma, Manassas, VA, USA) were maintained at 37 °C with 5% CO_2_ (standard culture conditions) in Dulbecco’s Modified Eagle’s Medium (DMEM, Gibco, Carlsbad, CA, USA) supplemented with 7% fetal bovine serum (FBS, Gibco/Invitrogen, Carlsbad, CA, USA), 100 U/mL penicillin, and 100 μg/mL streptomycin (Gibco, Carlsbad, CA, USA). SKOV3 cells (ATCC) were maintained under standard culture conditions in Advance Roswell Park Memorial Institute (RPMI) 1640 medium (Gibco, Carlsbad, CA, USA) supplemented with 10% FBS, 100 U/mL penicillin, and 100 μg/mL streptomycin (Gibco, Carlsbad, CA, USA).

### 2.4. Plasmid Purification and Transfection

Purification and transfection of pAm-Cyan and pAm-Cyan-LC plasmids were performed as previously described [4]. Briefly, pAmCyan1-N1 (Clontech, Cardiff, UK) eukaryotic expression vector and pAmCyan1-N1-LC with the LC sequence from FCV Urbana strain, previously obtained by our workgroup [4], were used to transform chemically competent *E. coli* DH5α cells. Plasmid was extracted with Jena Bioscience Fast-n-Easy maxi prep kit. CrFK and SKOV3 cells were grown to 80% confluence and transfected with Lipofectamine 2000 (Invitrogen, Carlsbad, CA, USA) following manufacturer instructions for 48 h, and proteins were extracted as previously described for Western blot analysis.

### 2.5. Recombinant Protein Expression and Purification

Chemically competent E. coli BL21 cells were transformed with the pRSETA-Histag-LC plasmid and cultured in Luria Broth (1.0% Tryptone *w*/*v*, 0.5% Yeast Extract *w*/*v*, 1.0% NaCl *w*/*v*) as previously described [5]. Once the culture reached an optical density of 0.4 at a wavelength of 600 nm, protein expression was induced by adding Isopropyl-ß-D-thiogalactopyranoside (IPTG) (ThermoFisher, Waltham, MA, USA) at a final concentration of 1 mM. Proteins from the inclusion body fraction of transformed cells were obtained according to the QIAExpressionist (Qiagen, Venlo, The Netherlands) 9 and 10 protocols with 8M urea (Invitrogen, Carlsbad, NM, USA). According to the manufacturer’s instructions, the recombinant protein was further purified through Fast Protein Liquid Chromatography (FPLC) using a Ni-NTA column (Thermofisher, Waltham, MA, USA). Eluted fractions in elution buffer (300 mM imidazole, 50 mM NaH_2_PO_4_, 300 mM NaCl pH 8.0) were analyzed through SDS-PAGE, and the gel was stained with coomassie blue to determine the integrity and purity of the Histag-LC protein. Selected fractions were quantified using the bromophenol blue method and used for the in vitro exogenous interaction and cell permeation assays.

### 2.6. In Vitro Exogenous Interaction of the LC Protein with the Plasmatic Membrane of CrFK Cells

CrFK cells grown on coverslips were interacted with 1 μM of the recombinant LC protein from FCV or the recombinant NS1 protein from ZIKV (Aalto Bio Reagents, Dublin, Ireland, ROI) as a control, in DMEM for 3 h. The cells were washed with PBS and fixed with 3.7% paraformaldehyde (*v*/*v*) in PBS for 20 min. Blocking was performed with 0.5% porcine skin gelatin for 30 min, followed by overnight incubation with the anti-LC and anti-flavivirus NS1 (Abcam, Cambridge, UK) in PBS, as indicated. Secondary Alexa 488 (Thermofisher Scientific, Waltham, MA, USA) was diluted 1:80 in PBS and incubated 2 h at room temperature. The cells were stained with 4′,6-Diamidino-2-phenylindole dihydrochloride (DAPI) (Thermofisher Scientific, Waltham, MA, USA) in PBS for 5 min. Mounting was performed with VectaShield (Vector laboratories, Newark, NJ, USA), and analysis was performed using a Zeiss LSM 900 confocal microscope with ZEN lite 2.5 software. All images were taken as optical sections on the *z*-axis.

### 2.7. Cell Permeation Assays

All cell lines (CrFK, SKOV3, and ID8) were grown in 96-well plates to a 100% confluence and incubated with indicated concentrations of the previously purified Histag-LC or Histag-LC-C40A recombinant proteins at the indicated times. Sytox green reagent was used at a final concentration of 0.5 mM to verify cell membrane permeability. Sytox green-positive cells were determined by epifluorescence using a Nikon TE series inverted microscope. Total well fluorescence was determined every 15 min using the Thermo Fluoroskan Ascent FL. For reduced protein assays, the Histag-LC protein was treated with DTT 30 mM for 30 min before interaction with cells at a final DTT concentration of 7.5 mM.

### 2.8. SDS-PAGE Western Blot Analysis

Total protein extracts from CrFK, SKOV3, and ID8 cells obtained with RIPA lysis buffer (150 mM NaCl, 1% Nonidet N-P40, 0.5% deoxycholate, 0.1% SDS, and 50mM Tris-HCl pH 7.4) and were quantified using the Pierce BCA Protein Assay Kit (Thermo Scientific, Waltham, MA, USA. An amount of 10 μg of total protein extracts were analyzed by SDS-PAGE and transferred to a nitrocellulose membrane. The membrane was blocked with 5% skimmed milk for 30 min and incubated overnight at 4 °C with anti-survivin (Cell Signaling Technology, Danvers, MA, USA), anti-Cyan (Clontech, Cardiff, UK), anti-procaspase, and anti-GAPDH (Santa Cruz Biotechnology, Dallas, TX, USA) antibodies. Membranes were washed with PBS and incubated with anti-rabbit (for survivin) and anti-mouse (for GAPDH) HRP-conjugated secondary antibodies (Jackson Immunoresearch, West Grove, WI, USA). The signal was detected using an autoradiography film and analyzed using the ImageJ software (http:/rsb.info.nih.gov/ij accessed on 23 June 2024).

## 3. Results

### 3.1. Validation of the Predicted Tertiary Structure of the Wild Type and the C40A Mutant LC Proteins from the FCV

**Figure 1:**
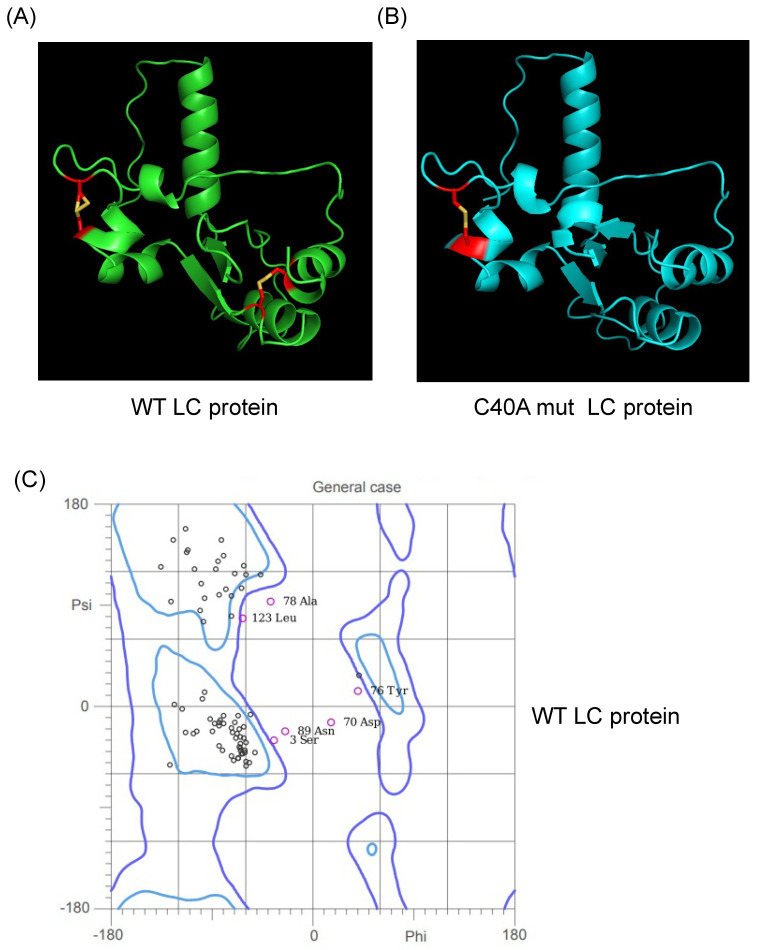

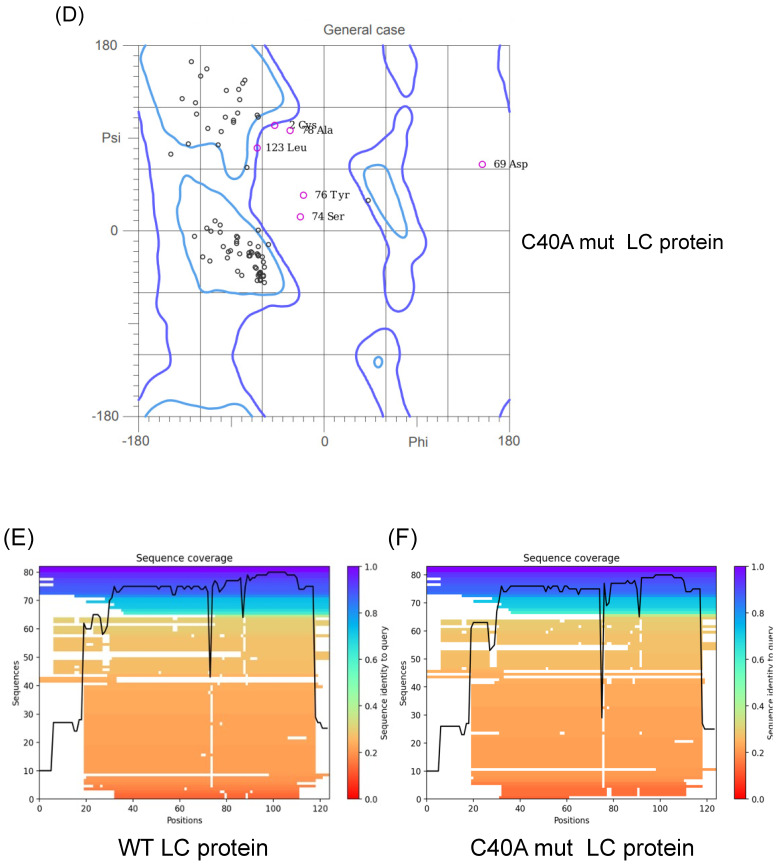

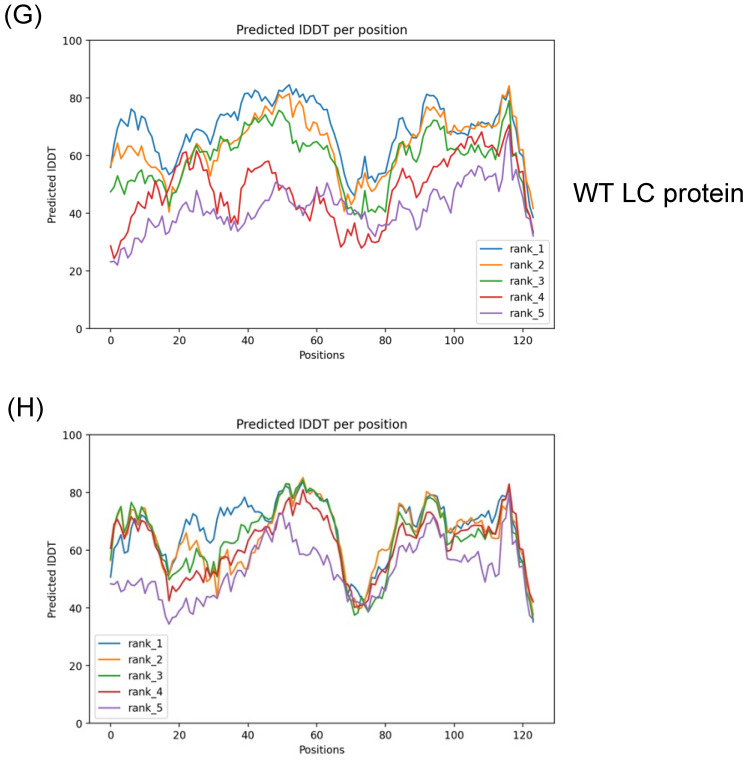
Tertiary structure modeling of the wild type and C40A mutant FCV LC proteins using the AlphaFold 2 predictor. Cartoon of the predicted model of the (**A**) WT (green) and (**B**) C40A mut (blue) LC proteins from FCV with the highest pLDDT score. Intramolecular disulfide bonds are highlighted in red, with sulfur atoms marked in yellow. Ramachandran plot of the highest score of the predicted models of the (**C**) WT and (**D**) C40A mut LC proteins. Coverage of the predicted models of the (**E**) WT and (**F**) C40A mut LC proteins, with the heatmap indicating the identity of the aligned sequences for each residue (blue indicates an identity of 1.0) and, in the *y*-axis, the number of sequences matched for each residue. plDDT of the predicted models of the (**G**) WT and (**H**) C40A mut LC proteins with AlphaFold 2.

The previous predictions of the tertiary structure of the FCV LC protein using a standard homology-based software yielded models with low confidence scores, with several amino acid residue φ/ψ angle values in non-favorable or forbidden positions in a Ramachandran analysis (Figure S1A). We predicted their putative tertiary structure by using the recently released AlphaFold 2 collaboration notebook to model both wild-type (WT) and C40A mut LC proteins (Figure 1A,B). Ramachandran plots of the highest score of the WT and C40A mut LC proteins were also predicted (Figure 1C,D). The predicted models do not have high coverage (Figure 1E,F) compared to other modeled proteins due to their previously reported low homology [3]. However, at least one model per protein has an acceptable pIDDT score for both the WT and C40A mut LC proteins (Figure 1G,H). Evaluation of the higher pIDDT score models with MolProbity [10] showed that there is an overall acceptable Z-score and that the majority of the φ/ψ angles of each residue falls under favorable or accepted values (Figure 1G,H) for both WT and C40A mut LC proteins when compared to the predicted tertiary structure model of the WT protein by I-Tasser QUARK (Figure S1B).

Therefore, these predicted tertiary structure models for the WT and C40A mut LC proteins from FCV (Figure 1A,B) can be used for further bioinformatic analysis. While carrying out these assays, the AlphaFold 2 predictor was upgraded to AlphaFold 3. However, we have not observed substantial differences between the predictions from these two versions (Figure S2).

### 3.2. Putative Wild-Type and C40A Mutant LC Protein Tertiary Structures Possess Intramolecular Disulfide Bonds in Accordance with Experimental Data

During our in vitro characterization of the FCV LC protein, we determined that both the WT and the C40A mut proteins can homo-oligomerize through disulfide bond formation [5]. We also determined that both proteins form intramolecular disulfide bonds through in vitro assays where a shift in migration of the monomeric form under non-reducing conditions was observed [5]. Nevertheless, these experimental results could not be correlated to the analysis of the intramolecular disulfide bond formation found by Disulfind software (http://disulfind.dsi.unifi.it/ accessed on 23 June 2024) [12], in which the C40A mut LC protein did not show the formation of any intramolecular disulfide bond. However, using AlphaFold 2 software, we found that both the WT and the C40A mut proteins present putative intramolecular disulfide bonds. The WT LC protein showed intramolecular disulfide bonds between cysteine residues 5–54 and 33–40; the latter is contained in the CR-I (Figure 1A, highlighted in red) and distinct from the 2–33, 5–39, and 40–54 residues previously predicted with the Disulfind [5]. On the other hand, the putative tertiary structure of the C40A mut LC protein obtained using the AlphaFold 2 showed one predicted intramolecular disulfide bond, in concordance with the previous experimental results published by our workgroup, between cysteine residues 33 and 39 (Figure 1B, highlighted in red). All these results, taken together, strongly suggest that both the WT and C40A mut LC proteins from FCV form intramolecular disulfide bonds. Moreover, the results concurrency with experimental data validates the tertiary structure predictions by AlphaFold 2 algorithms.

### 3.3. The FCV LC Protein Predicted Structure Lacks a Defensin γ-Core Signature in Its CR-I But Has a Putative C-Terminal Transmembrane Domain

Our workgroup reported that the FCV LC protein probably lacks a TMD but has in its CR-I the GXCX_3–9_C sequence, the molecular signature γ-core of defensins, a subgroup of antimicrobial peptides [13]. After modeling the tertiary structure of the LC CR-I sequence, we found that it maintains the typical structure of this defensins’ signature, suggesting that the LC protein might function as a viroporin that relies on disulfide bonds [5], similar to the Delta peptide present in the *Ebola virus* (EBOV) [14]. The tertiary structure of the FCV LC protein predicted by AlphaFold 2 does not contain the spatial distribution of the γ-core signature when compared with defensins that had this structure determined experimentally, such as the Human Beta Defensin 1 [13,15], suggesting that the LC viroporin activity is not due to its similarity to an antimicrobial peptide as previously thought.

**Figure 2:**
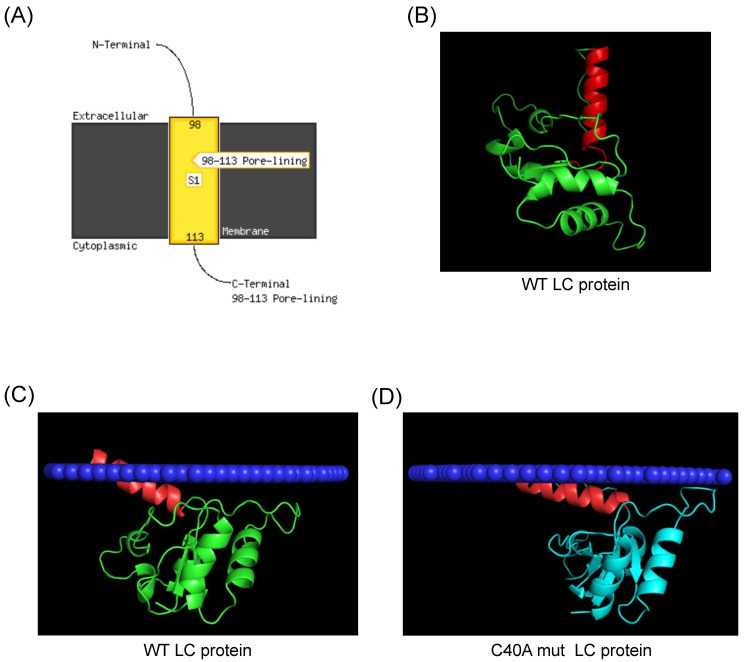
Prediction of a C-terminal transmembrane domain of the FCV LC protein. (**A**) TMD prediction of the FCV LC protein sequence with PSI-Pred server. (**B**) Predicted tertiary structure of the FCV LC protein (green) with AlphaFold 2, with the putative C-terminal region highlighted in red. (**C**) Prediction of the WT (green) and (**D**) C40A mut (blue) LC proteins C-terminal TMD insertion in a mammalian membrane (dark blue) with PPM3, with the putative TMD of the LC protein highlighted in red.

We predicted the tertiary structure with the most recent software to determine if the FCV LC protein could have a TMD. We analyzed it with AlphaFold 2 to determine if it correlates with this updated information (Figure 2). We found that the FCV LC protein has a putative TMD in its C-terminal region, spanning between amino acids 98–113 when its sequence was analyzed with the PSI-PRED TMD predictor (Figure 2A). These results, obtained with improved bioinformatic tools, suggest that the FCV LC protein could be a class-I viroporin, marked by a single TMD with which it could exert its pore or channel forming function.

The analysis of the predicted 3D structure of the FCV LC protein obtained with AlphaFold 2 shows that there is an alpha helix structure in the C-terminal domain of the protein (Figure 2B, highlighted in red), spanning the same amino acid residues (98–113) that are predicted to be in the TMD (Figure 2A). To hypothesize further if this C-terminal region can be inserted into membranes, we used the molecular dynamics server PPM 3.0, which measures the energy transferred from a protein when inserted into a phospholipid membrane of a determined composition [11]. We found that both the FCV WT and C40A mut LC proteins’ tertiary structures have a favorable prediction of insertion into a mammalian cytoplasmic membrane through its C-terminal helix (Figure 2C,D). We also found that both proteins have a distinct angle of insertion into the phospholipid bilayer (Figure 2C,D). These results altogether strongly suggest the presence of a TMD in the C-terminal region of the FCV LC protein.

### 3.4. Modeling of FCV Wild Type and C40A Mutant LC Protein Oligomeric Forms by AlphaFold 2

**Figure 3:**
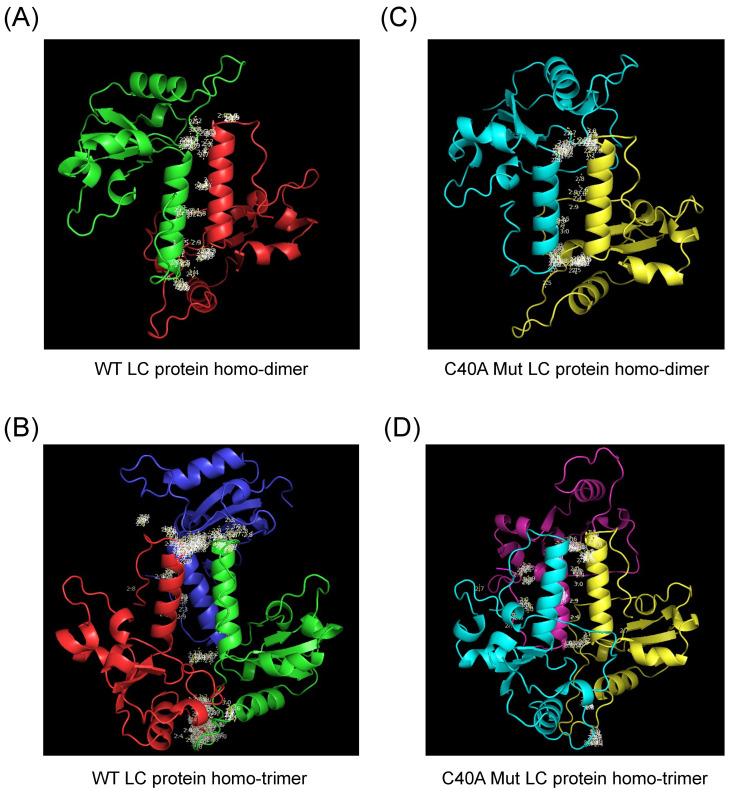
Modeling of the homo-oligomeric forms of the FCV WT and C40A mutant LC proteins using AlphaFold 2. Cartoon representation of the tertiary structure prediction of the (**A**) WT homo-dimer (green and red), (**B**) WT homo-trimer (green, red, and dark blue), the (**C**) C40A mut homo-dimer (blue and yellow), or (**D**) C40A mut homo-trimer (blue, yellow, and magenta) of the FCV LC proteins with AlphaFold 2. Amino acid interactions between chains are highlighted in white.

We have previously reported that the FCV LC protein can homo-oligomerize through disulfide bond formation; however, whether these bonds are intermolecular, intramolecular, or both was not determined. By using the recently developed oligomer option of AlphaFold 2 [16] to determine if there are possible homo-dimer and homo-trimer arrangements of the WT and C40A mut FCV LC proteins, we found that there is one arrangement that has an acceptable pLDDT score for the homo-dimeric and homo-trimeric forms (Figure 3A and 3B, respectively). In both cases, the highest proximity between residues in the protein changes is in the putative C-terminal TMD previously identified in the monomeric form, which suggests that this domain could mediate its oligomerization. Interestingly, the modeling of the homo-dimeric and homo-trimeric forms of the C40A mut LC protein predicted more than one possible conformation (Figure S3), with the higher score models also showing proximity between putative TMDs (Figure 3C,D).

The predicted homo-dimeric form of the WT LC protein has no intramolecular nor intermolecular disulfide bonds. In contrast, the homo-trimeric arrangement prediction has only one disulfide bond between the cysteine residues 5 and 56 of the same amino acid chain (Figure S4A,B). The C40A mut LC homo-dimeric and homo-trimeric forms present no changes in intramolecular disulfide bond formation when compared to the monomer (Figure S4C,D), with the intramolecular disulfide bond between 33 and 39 cysteine residues being present in the three models, in discordance with our previous results in which LC oligomers were dependent on disulfide bond formation [5].

### 3.5. The Purified Histag-LC Protein from FCV Associates with the CrFK Cells’ Cytoplasmic Membrane through Exogenous Interaction

**Figure 4:**
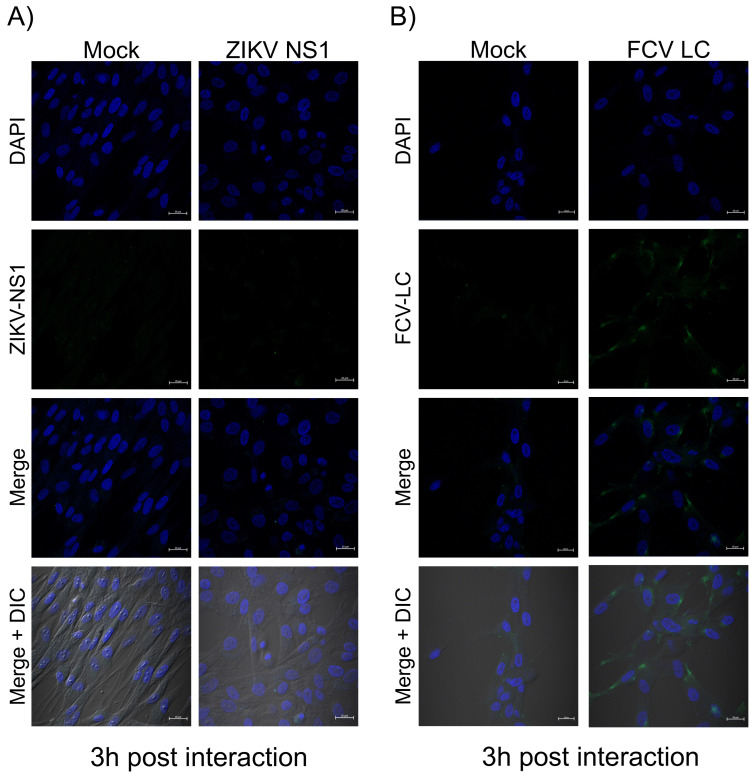
The purified Histag-LC protein from FCV is associated exogenously with the cytoplasmic membrane of CrFK cells. Monolayers of CrFK cells interected or not (mock) with 1 μM of the (**A**) Histaged-LC or (**B**) ZIKV NS1 proteins were immunostained for 3 h with an anti-LC serum or the anti-flavivirus NS1 antibodies (Green). DAPI was used for nuclear (blue) staining. The cells were examined in a Zeiss LSM 700 confocal microscope. The images correspond to a z-stack of 15 slices and represent two independent experiments. Merged images are indicated.

Since the LC protein tertiary structures analyzed above showed a favorable prediction of insertion into mammalian cytoplasmic membranes and a putative TMD in the C-terminal region of the protein, we wanted to determine if it could in vitro associate with the cytoplasmic membrane. Therefore, monolayers of CrFK cells were interacted with 1 μM of the purified Histag-LC protein or 1 μM of the ZIKV NS1 protein (as a negative control) for 3 h, and its presence in the cytoplasmic membrane of non-permeabilized cells was determined by immunofluorescence assays using a confocal microscope (Figure 4). The ZIKV NS1 protein has been previously reported to be internalized in mammalian and insect cells after 1 or 2 h of exogenous incubation [17]; thus, it was used as a negative control (Figure 4A). No signal was observed in the surface of the non-permeabilized cells that interacted with the ZIKV NS1 protein or in the mock conditions (Figure 4A,B). In contrast, a green signal on the surface of the cells that interacted with the Histag-LC protein was observed as patches (Figure 4B), indicating its association with the cell membrane.

### 3.6. The Purified Histag-LC Protein from FCV Permeates CrFK Cells’ Cytoplasmic Membrane through Exogenous Interaction and in a Disulfide Bond-Independent Formation

**Figure 5:**
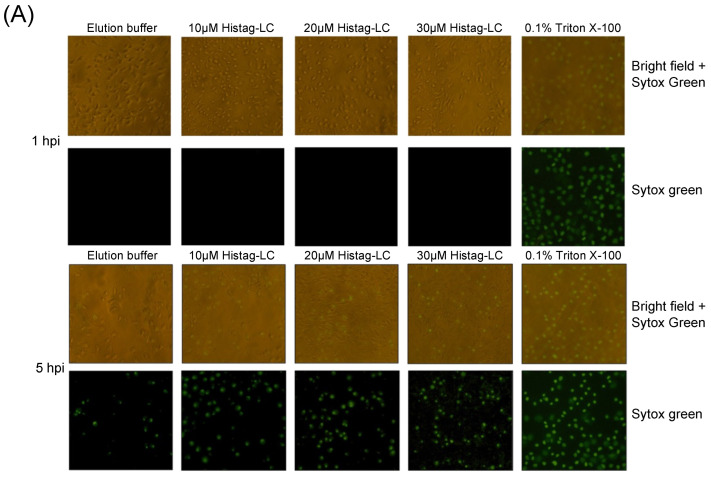

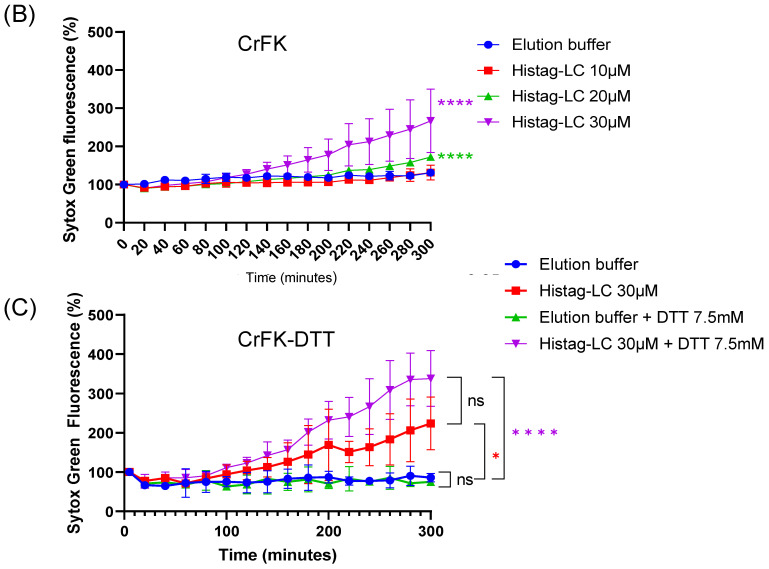
CrFK cells permeate the cytoplasmic membrane through the exogenous interaction with the Histag-LC protein. (**A**) Representative images of CrFK cells incubated with elution buffer or 10, 20, and 30 mM of the WT Histag-LC LC protein, obtained using epifluorescence microscopy. As a positive control, 0.1% Triton X-100 was used. Sytox green dye at 0 and 5 hpi is shown. Total well plate fluorescence percentage of CrFK cells incubated with (**B**) 10, 20, and 30 mM of the Histag-LC protein in the presence of Sytox green or (**C**) 30 mM of the Histag-LC protein previously reduced with 30 mM DTT and in the presence of a final concentration of 7.5 mM DTT and Sytox green at the indicated times, obtained using a Fluoroskan Ascent FL. Standard deviations were obtained from three independent experiments. Non-significant (ns) differences are indicated. Values of *p* < 0.01 and (*) *p* < 0.0001 (****) were calculated by T-test using GraphPad Prism 8.0 software and are indicated.

Since the previous predictions of the FCV LC protein’s tertiary structure suggest that its viroporin characteristic is mediated through a C-terminal TMD and the exogenous in vitro interaction indicates the association of the LC protein with the surface of non-permeabilized cells, we wanted to determine its capability to permeate cytoplasmic membranes through an exogenous interaction. To this end, increasing concentrations of a Histag-LC purified protein were incubated with a monolayer of CrFK cells in the presence of Sytox green, a fluorescent DNA-binding dye that cannot permeate healthy membranes and has been previously used to assess viroporin activity [14] (Figure 5). We found that there is a time and dose-dependent increase (10, 20, and 30 μM) in Sytox green-positive CrFK cells by epifluorescence microscopy (Figure 5A) as well as by measuring the total well fluorescence by fluorescence scanning (Figure 5B). To determine if the membrane permeation induced by the WT Histag-LC protein was dependent on its disulfide bonds, the recombinant Histag-LC protein was reduced by DTT treatment prior to its interaction with the cells (Figure 5C).

The Sytox green entry assays performed with 30 μM of the 7.5 μM of DTT-reduced WT Histag-LC protein showed no significant reduction in its permeation ability in comparison to the observed with the untreated non-reduced WT Histag-LC protein (Figure 5B,C). These findings indicate that the WT recombinant LC protein can permeate mammalian membranes through exogenous interaction, a common viroporin characteristic, and that this activity does not depend on disulfide bond formation.

### 3.7. The Purified Histag-LC Protein from FCV Permeates the Cytoplasmic Membrane of the Human SKOV3 and the Murine ID8 Ovarian Cancer Cells through an Exogenous Interaction

**Figure 6:**
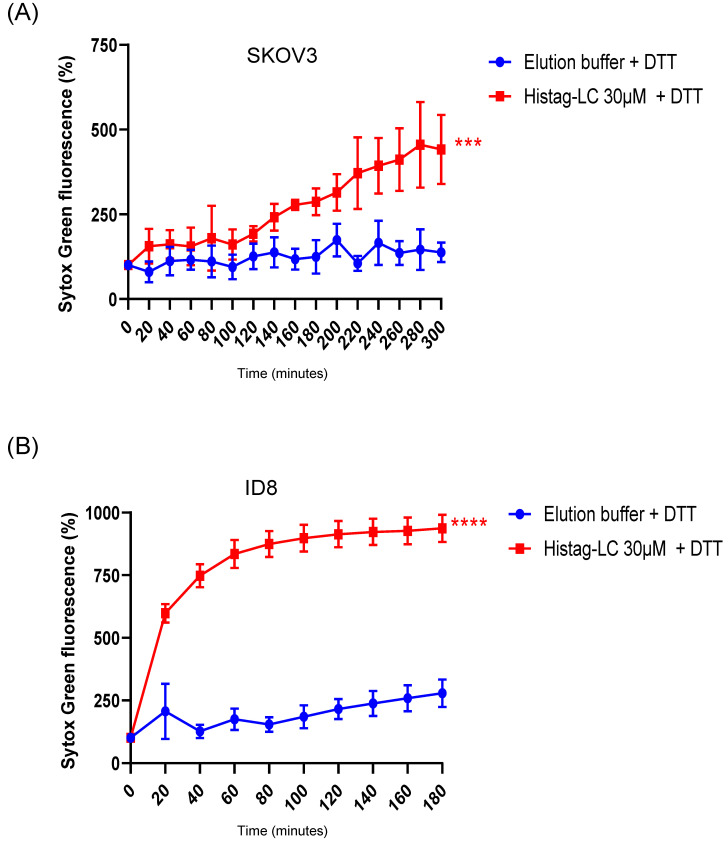
Permeation of SKOV3 and ID8 ovarian cancer cell lines with FCV LC protein. Total well plate fluorescence percentage of (**A**) SKOV3 and (**B**) ID8 cells incubated with 30µM of the Histag-LC protein in the presence of 5mM DTT and Sytox green at the indicated times, obtained using a Fluoroskan Ascent FL. Standard deviations were obtained from three independent experiments. Values of *p* < 0.001 (***) and *p* < 0.0001 (****) were calculated by *T*-test using GraphPad Prism 8.0 software and are indicated.

It is well known that cells that undergo cancerous transformation experience changes in the lipid composition of their cytoplasmic membranes, with altered fluidity, motility, and molecule exchange [reviewed in [18,19]]. Therefore, we aimed to determine if the FCV LC protein could permeate the cytoplasmic membrane of ovarian cancer cell lines through an exogenous interaction (Figure 6).

As with CrFK cells, the exogenous interaction of the reduced-Histag-LC protein with cancer cells caused permeation of both SKOV3 (human) and ID8 (murine) ovarian cancer cell cytoplasmic membranes (Figure 6A and Figure 6B, respectively), with the ID8 cells being more susceptible to the interaction than the SKOV3 and the CrFK cells, which suggests that this protein induces cytoplasmic membrane damage in different cells.

### 3.8. The FCV LC Protein Expression in a Virus-Free System Induces Apoptosis in SKOV3 Cells

Apoptosis through the intrinsic pathway is commonly triggered when cells undergo mutations that alter their cell cycle to eliminate unnecessary or abnormal cells [reviewed in [20]]. Therefore, it is not surprising that changes in the pro-apoptotic and anti-apoptotic gene expression play a key role in the transformation and carcinogenesis process [21]. For example, the anti-apoptotic proteins survivin and XIAP, members of the IAP family, are overexpressed in cancer cells and correlate with poor prognosis in cancers treated with some of the most used chemical agents, such as cisplatin [22].

As previously reported by our workgroup, both FCV infection and LC protein sole expression in CrFK cells resulted in the degradation of survivin and XIAP as part of the apoptosis onset [4]. Therefore, we wanted to test if the LC protein expression alone was sufficient to induce apoptosis in ovarian cancer cell lines that overexpress survivin (Figure S5A,B).

**Figure 7:**
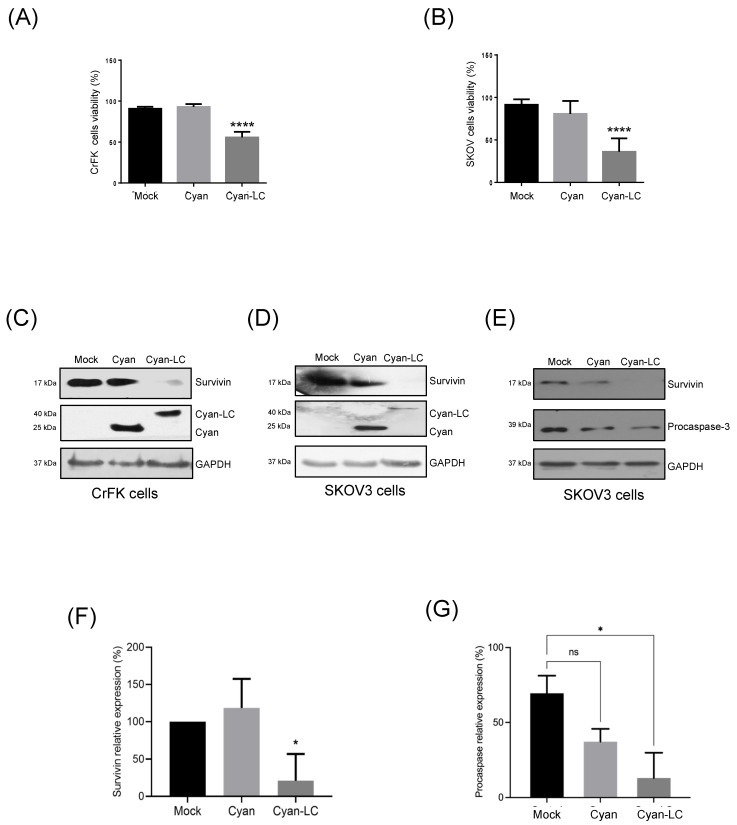
Reduction in cell viability and induction of apoptosis in SKOV3 ovarian cancer cell lines expressing the FCV LC protein. Cell viability percentage of mock-transfected and pAm-Cyan and pAm-Cyan-LC-transfected (**A**) CrFK and (**B**) SKOV3 cells for 48 h. Total extracts from transfected (**C**) CrFK (left panel) and (**D**) SKOV3 cells (right panel) were obtained and assessed for Cyan, Cyan-LC, and survivin expression by Western blot. (**E**) Total extracts from transfected SKOV3 cells were obtained and assessed for survivin and procaspase expression by Western blot. Relative expression levels of (**F**) survivin and (**G**) procaspase. Densitometric values were obtained using FUJI software. (https://www.fujifilm.com accessed on 23 June 2024). Standard deviations were obtained from three independent experiments. Non-significant (ns) differences are indicated. Values of *p* < 0.05 (*) and *p* < 0.01 (****) were calculated by two-way ANOVA using GraphPad Prism 10.0 software and are indicated.

To determine if the expression of the LC protein is associated with the downregulation of survivin and apoptosis induction in transfected SKOV3 cells, monolayers were transfected with both the pAm-Cyan and the Wt-LC-pAm-Cyan plasmids, and the CPE was evaluated by epifluorescence microscopy as previously described for CrFK cells [4]. The expression of the LC protein in SKOV3 cells caused a characteristic CPE, such as cell rounding and disruption of the monolayer, in comparison to the transfected cells with the empty vector pAm-Cyan where the monolayer remained confluent and most of the cells had typical morphology (Figure S5C). The viability and survivin integrity from CrFK- and SKOV3-transfected cells with both pAm-Cyan and Wt-LC-pAm-Cyan cells were analyzed by MTT assays and Western blotting (Figure 7). A strong reduction of approximately 45% and 60% in cell viability was observed when the LC protein was expressed in CrFK and SKOV3 cells, respectively (Figure 7A,B). Moreover, a significant reduction of 75% in survivin protein relative levels was observed in total protein extracts from SKOV3 (Figure 7D,F), as was previously described in CrFK cells (Figure 7C) [4], indicating that the expression of the LC protein also causes a reduction in the survivin levels in an ovarian cancer cell line that overexpresses survivin. Moreover, a reduction in procaspase levels from SKOV3 cells (Figure 7E,G) correlates with the reduction in survivin and strongly suggests that the LC protein can induce apoptosis in these cells. Taken together, these results indicate that the LC protein can trigger cell death in CrFK cells as well as in cell lines where apoptosis is impaired due to survivin overexpression.

## 4. Discussion

The role of the FCV LC protein in inducing the CPE during infection has been well documented [3], and our workgroup has determined some of its biochemical characteristics [5]. However, its precise role during infection is not fully understood. We have previously reported that the FCV LC protein can induce apoptosis through the intrinsic pathway when expressed in a virus-free system [4]. It also exhibits key viroporin characteristics such as homo-oligomerization and toxicity induction through osmotic stress [5].

The subcellular localization, as well as the possible roles of the LC protein during infection, are currently being studied [6]. Our first bioinformatics analysis in 2022 revealed that the FCV LC protein lacked a TMD [5]; however, a recent revisiting of TMD prediction servers shows a putative C-terminal TMD in this protein. Furthermore, the AlphaFold 2 prediction of its tertiary structure and the in silico insertion of this 3D model into a plasma membrane suggest that the FCV LC protein has a single TMD. The in vitro interaction of the recombinant LC protein with CrFK cells’ surface and the ability to permeate these cells through an exogenous interaction correlates with these in silico findings.

The permeation of cellular membranes by LC protein exogenous interaction with mammalian cells, together with our recent work that determined the presence of LC protein in the extracellular medium of FCV-infected cells [6], reinforces the hypothesis that the LC protein is a viroporin that is secreted during virus replication, with possible toxin and pathogenesis functions.

Interestingly, the unaffected permeation capability of this protein after reduction with DTT indicates that this interaction might depend on the TMD and not on disulfide bond formation, as we previously proposed [5]. The in silico analysis of the LC proteins from different vesiviruses, such as *Canine calicivirus* (CaCV), *Vesicular exanthema of swine virus* (VESV), and *Mink calicivirus* (MCV), showed the presence of a putative TMD (Figure S6), suggesting its conservation and possible involvement in the protein function.

By using the Disulfind server [12], we were able to predict only intramolecular disulfide bonds in the WT LC protein; however, the AlphaFold 2 analysis of the predicted tertiary structure of both the WT and C40A mut LC proteins now showed intramolecular disulfide bonds, in concordance with the in vitro evidence from our workgroup [5]. Even though the cysteine at position 40 in the FCV LC protein was not responsible for the dimerization of the protein, two other cysteines in the CRI at positions 33 and 39 could be responsible for it. In this regard, substitutions in each cysteine were deleterious for virus growth, emphasizing its importance for protein function [3]. Oligomerization through disulfide bonds of the lagovirus non-structural protein p23, recently characterized as a viroporin, was determined by direct mutagenesis that allowed us to determine the precise cysteine responsible for its dimerization [23]. Thus, further investigation of the role of the C33 and C39 from the FCV LC protein in forming disulfide bonds will be required.

Oligomerization through TMD of peptide chains has been previously reported, with models proposing putative mechanisms of how this occurs [24,25]. The AlphaFold 2 dimer and trimer predictions suggest that oligomerization of the WT and the C40A mut LC proteins may occur through their C-terminal TMD, with the C40A mut LC protein exhibiting more stable conformations. This finding correlates with the in vitro evidence from Western blot analysis of the WT and the C40A mut Histag-LC purified proteins [5]. However, finding no predicted intermolecular disulfide bonds in the oligomeric protein models was surprising. Moreover, the loss of both intramolecular disulfide bonds in the predicted dimer of the WT LC protein and the presence of only one of these bonds in the homo-trimeric form between different residues compared to the monomer could suggest Alpha Fold 2 limitations in the prediction of these types of bonds. Experimental confirmation of the disulfide bonds formed in the monomeric and homo-oligomeric LC protein arrangements needs to be conducted, and the impact of cysteine residue mutants on its structure and functions would shed light on the importance of the CRI of this protein to correctly perform its function(s).

Given the FCV LC protein’s activity causing membrane permeation of epithelial and ovarian cancer cell lines and inducing apoptosis in SKOV3, a cell line that overexpresses survivin, there is a promising potential for this viral protein to be exogenously expressed through various approaches, such as non-viral and viral vectors. The LC expression could lead to downregulating survivin levels and contribute to treating solid tumors or cisplatin-resistant cells. This exciting potential underscores the significant biomedical applications of this viroporin, offering hope for future cancer treatments.

Collectively, these results indicate that the FCV LC protein can in vitro interact with the cytoplasmic membrane, most likely through a TMD as predicted by in silico approaches. However, to confirm the presence of this TMD and classify the LC protein as a class-I viroporin, further in vitro assays will be needed. This underscores the critical importance of ongoing research in this field [26].

## Data Availability

No additional new data was created.

## Supplementary Materials

The following supporting information can be downloaded at: https://www.mdpi.com/article/10.3390/v16081319/s1, Figure S1: Tertiary structure modeling and validation of the FCV LC protein; Figure S2: Comparison of the tertiary structure modeling of the FCV LC protein using AlphaFold 2 and AlphaFold 3 predictors; Figure S3: Predicted lDDT of multimeric forms of the WT and C40A mutant LC proteins by Alpha Fold 2; Figure S4: Intramolecular disulfide bond in the homo-oligomeric forms of the FCV WT and C40A mutant LC proteins using AlphaFold2; Figure S5: Survivin expression levels in CrFK-, SKOV3-, and ID8-transfected cells; Figure S6: Prediction of a C-terminal transmembrane domain of the LC proteins from different vesiviruses.
