# Supplementary material for: The Feline calicivirus Leader of the Capsid (LC) Protein Contains a Putative Transmembrane Domain, Binds to the Cytoplasmic Membrane, and Exogenously Permeates Cells
Source: Viruses. 2024 Aug 19;16(8):1319. doi: 10.3390/v16081319 (PMC11359386; doi:10.3390/v16081319)
Supplement: Supplementary file 1 [file viruses-16-01319-s001.zip › viruses-3097996-supplementary.pdf]

Figure S1

A)

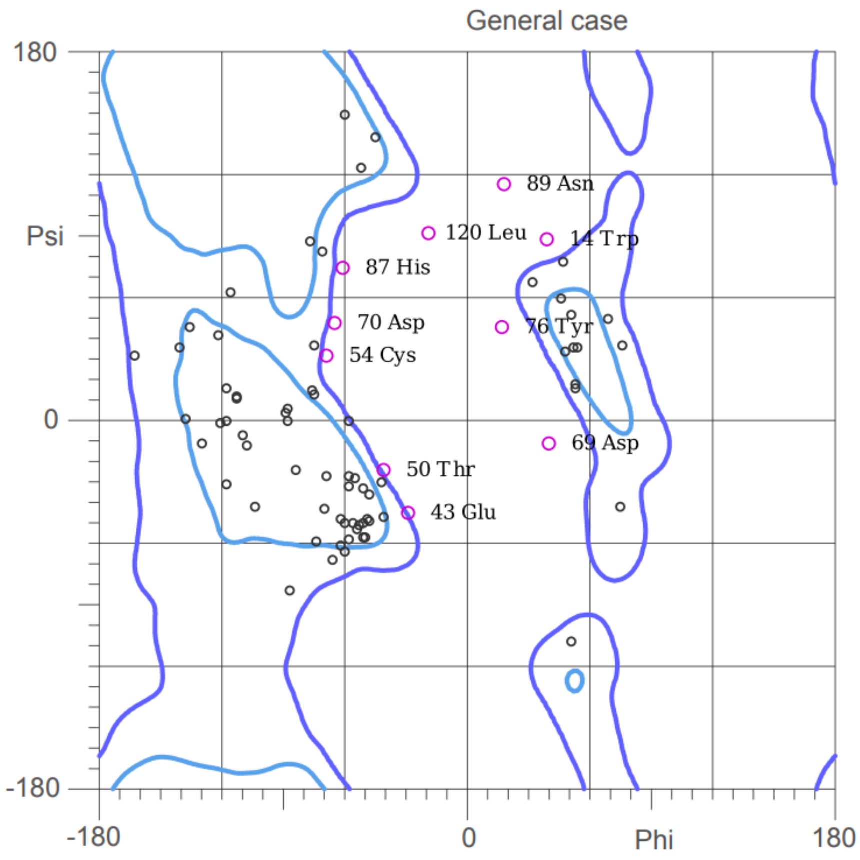
General case
180
Psi
0
-180
-180
0
Phi
180
89 Asn
120 Leu
14 Trp
87 His
70 Asp
76 Tyr
54 Cys
69 Asp
50 Thr
43 Glu

B)

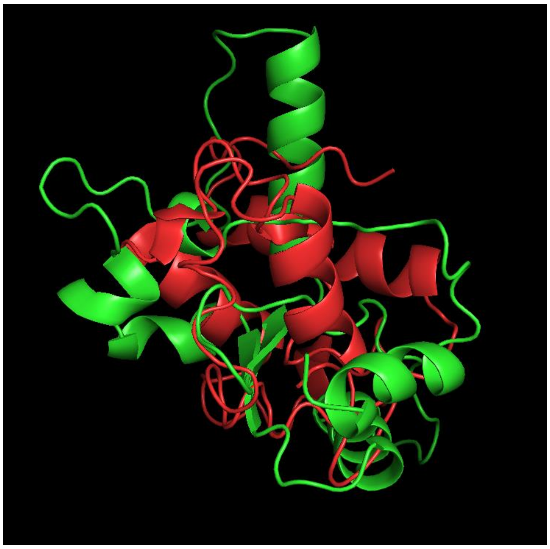

Figure S2

A)

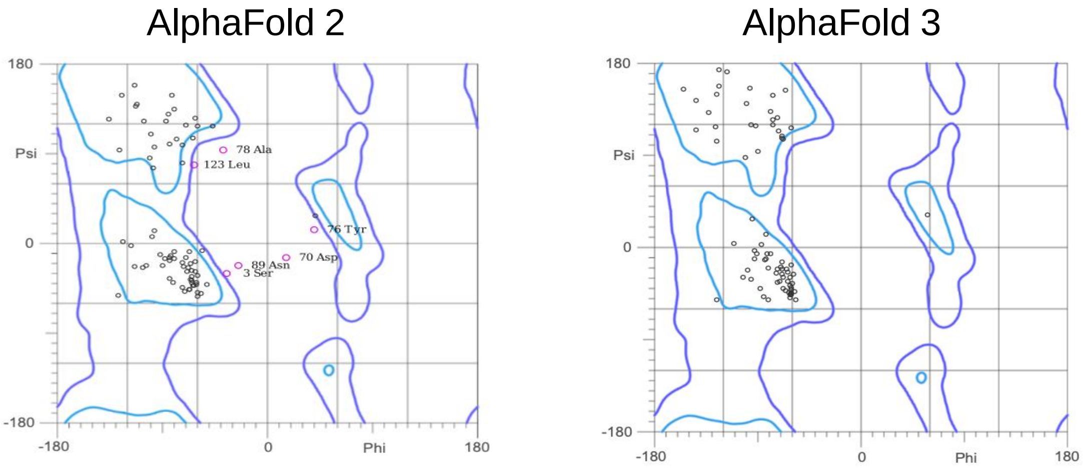

82.8% (101/122) of all residues were in favored (98%) regions.
92.6% (113/122) of all residues were in allowed (>99.8%) regions.

There were 9 outliers (phi, psi):
3 Ser (-35.5, -30.6)
31 Gly (-45.0, -12.7)
70 Asp (16.2, -14.8)
76 Tyr (40.7, 14.2)
78 Ala (-38.7, 94.8)
80 Asp (-14.8, 98.3)
81 Pro (-37.0, 111.4)
89 Asn (-25.4, -22.3)
123 Leu (-63.9, 79.9)

99.2% (121/122) of all residues were in favored (98%) regions.
100.0% (122/122) of all residues were in allowed (>99.8%) regions.

B)

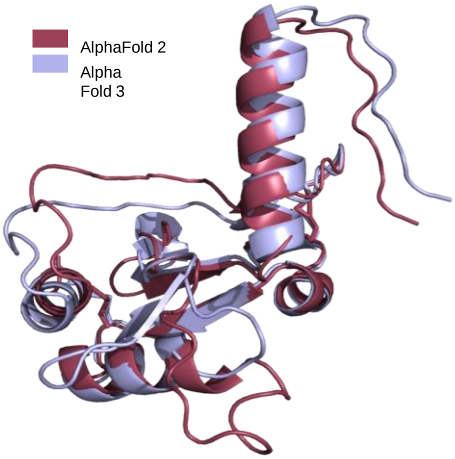

C)

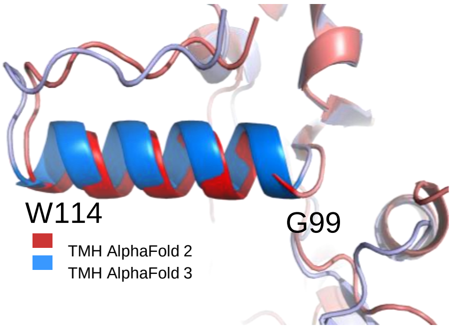

A)

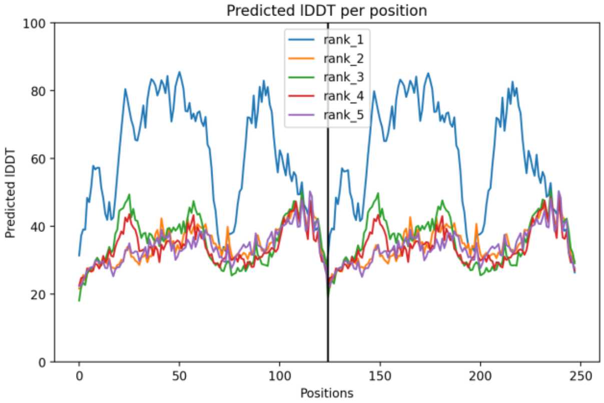

WT LC protein

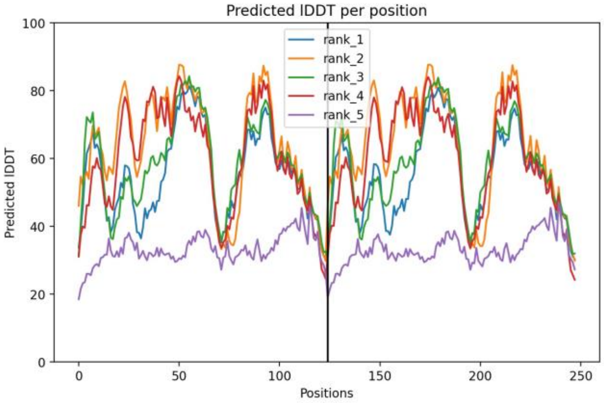

C40A Mut LC protein

B)

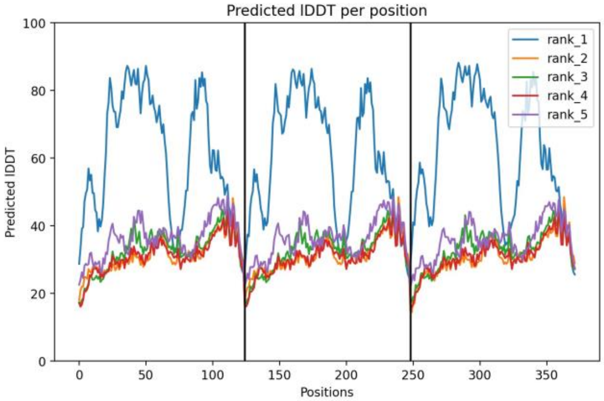

WT LC protein

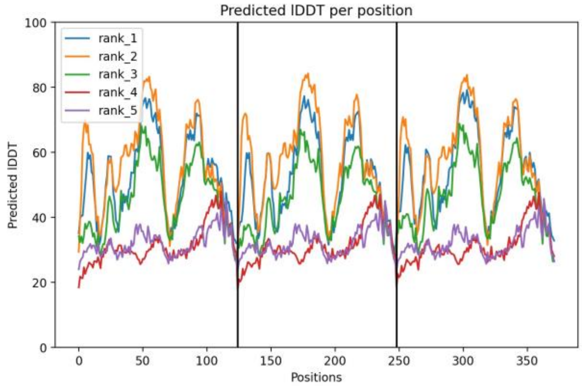

C40A Mut LC protein

Figure S4

A)

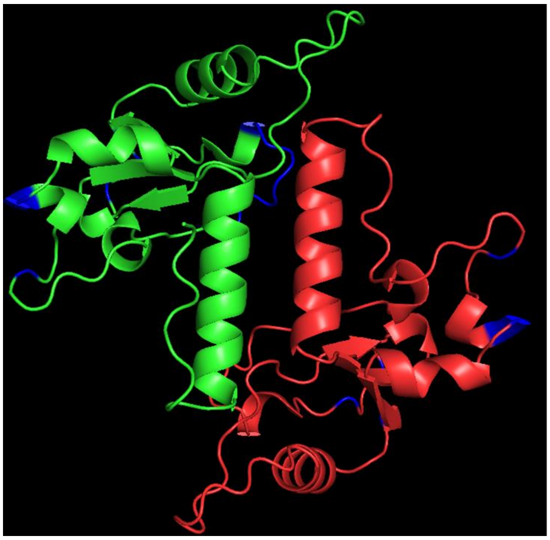

WT LC protein homo-dimer

C)

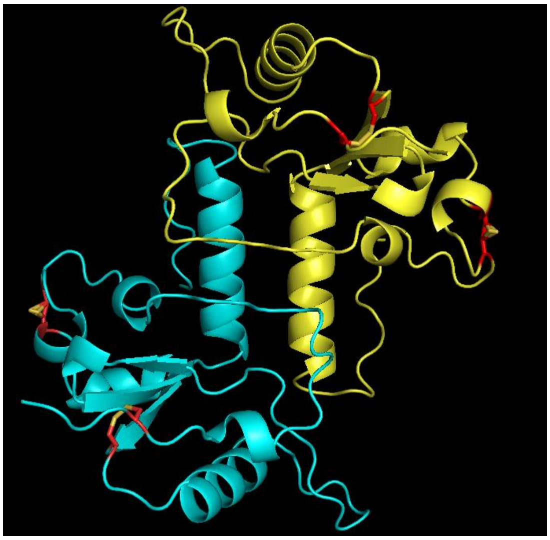

C40A Mut LC protein homo-dimer

B)

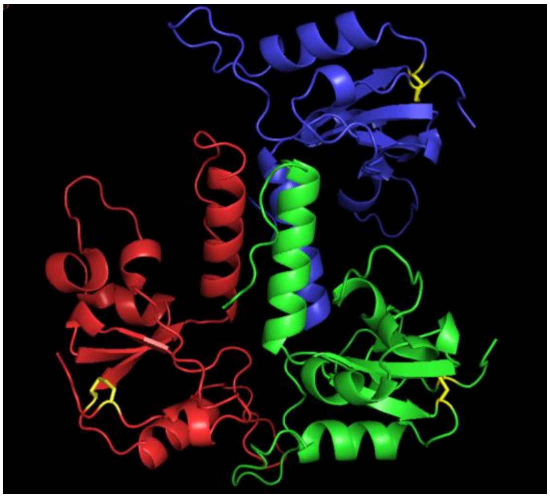

WT LC protein homo-trimer

D)

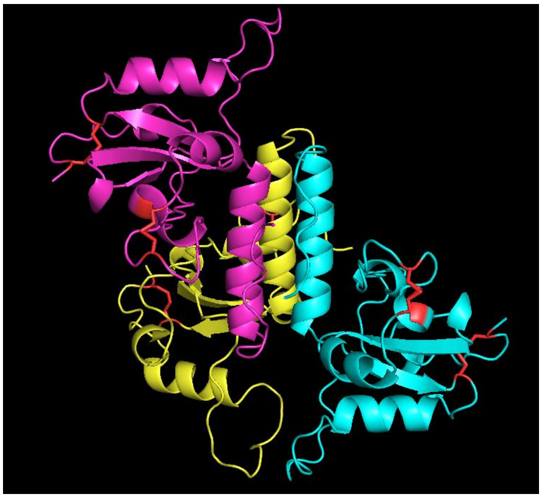

C40A Mut LC protein homo-trimer

LC wt

LC C40A

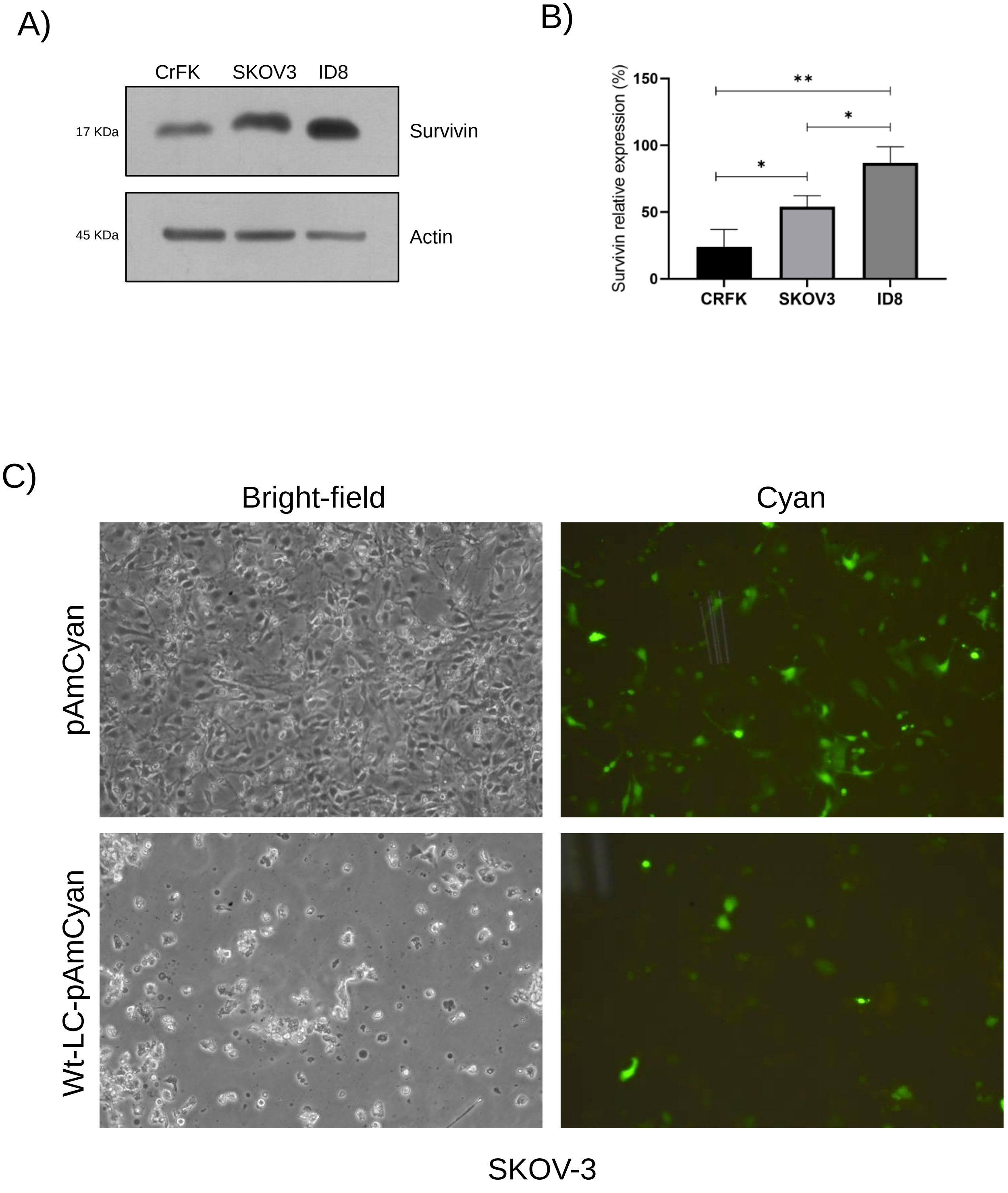
A)
CrFK
SKOV3
ID8
17 KDa
Survivin
45 KDa
Actin
B)
Survivin relative expression (%)
150
100
50
0
**
*
*
CRFK
SKOV3
ID8
C)
Bright-field
Cyan
pAmCyan
Wt-LC-pAmCyan
SKOV-3

Figure S6

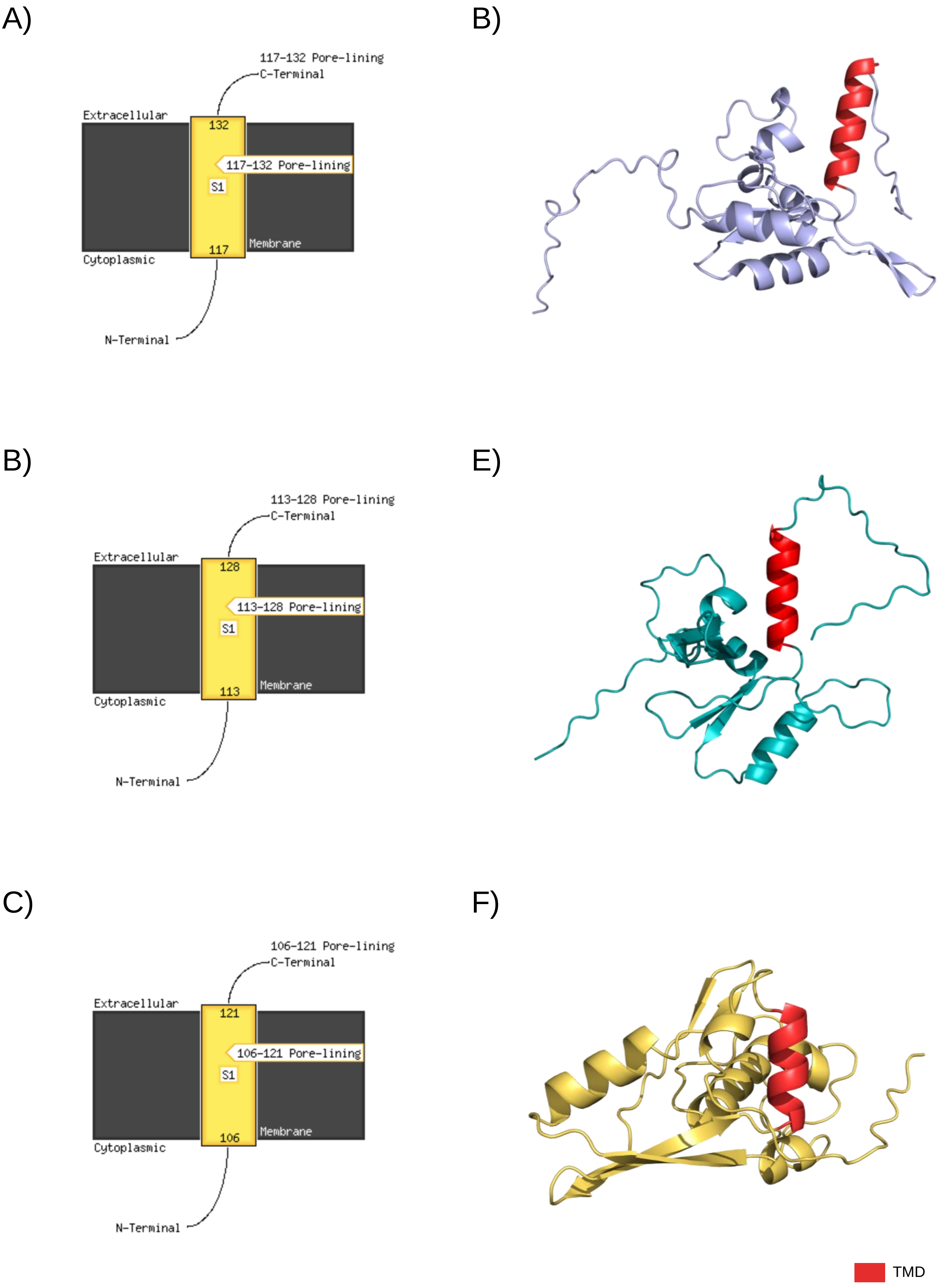
A)
117-132 Pore-lining
C-Terminal
Extracellular
132
117-132 Pore-lining
S1
117
Membrane
Cytoplasmic
N-Terminal
B)
B)
113-128 Pore-lining
C-Terminal
Extracellular
128
113-128 Pore-lining
S1
113
Membrane
Cytoplasmic
N-Terminal
E)
C)
106-121 Pore-lining
C-Terminal
Extracellular
121
106-121 Pore-lining
S1
106
Membrane
Cytoplasmic
N-Terminal
F)
TMD

Figure S1). Tertiary structure modeling and validation of the FCV LC protein. A) Ramachandran plot of the LC protein predicted models using Quark (I-Tisser, Zheng Labs). B) Alignment of the FCV LC protein tertiary structure from QUARK (red) and AlphaFold 2 (green).

Figure S2). Comparison of the tertiary structure modeling of the FCV LC protein using AlphaFold 2 and AlphaFold 3 predictors. A) Ramachandran plot of the highest score of the LC protein predicted models obtained using the AlphaFold 2 and AlphaFold 3 predictors. B) FCV LC protein and C) TMD domain models were obtained using the AlphaFold 2 (pink and red) and AlphaFold 3 (violet and blue) predictors, respectively.

Figure S3). Predicted lDDT of multimeric forms of LC by Alpha Fold 2. Predicted lDDT values along the sequences of A) dimers and B) trimers of the FCV wild type and C40A Mut LC proteins. The highest-ranked models were used for subsequent analysis.

Figure S4). Intramolecular disulfide bond in the homo-oligomeric forms of the FCV WT and C40A mutant LC proteins using AlphaFold2. Cartoon representation of the cysteine residues of the A) WT homo-dimer (dark blue), B) WT homo-trimer (yellow), C) C40A Mut homo-dimer (red), and D) C40A Mut homo-trimer (red) of the FCV LC protein with AlphaFold 2. The WT homo-dimer individual polypeptide chains are represented in green and red; the WT homo-trimer polypeptide chains in green, red, and dark blue; the C40A Mut homo-dimer polypeptide chains in blue and yellow; the C40A Mut homo-trimer polypeptide chains in blue, yellow, and pink.

Figure S5). Survivin expression levels in CrFK, SKOV3, and ID8 transfected cells. Total protein extracts from CrFK, SKOV3, and ID8 cells were obtained, and survivin levels were detected by western blotting. Actin was used as a loading control. Band intensities from scanned images were quantified using ImageJ software and shown as relative expression. Standard deviations were obtained from three independent experiments. Values of $p \leq 0.5$ (*) and $p \leq 0.005$ (**) were calculated by T-test using GraphPad Prism 8.0 software are indicated.

Figure S6). Prediction of a C-terminal transmembrane domain of the LC proteins from different vesiviruses. A TMD prediction of the A) canine calicivirus (CaCV), B) vesicular exanthema of swine virus (VESV), and C) mink calicivirus (MCV) LC protein sequences with PSI-Pred server. The tertiary structure prediction of the D) canine calicivirus (CaCV), E) vesicular exanthema of swine virus (VESV), and F) mink calicivirus (MCV) with AlphaFold 3, with the putative C-terminal region highlighted in red.
